# Knowledge of teachers about suicidal behavior in adolescents**

**DOI:** 10.17533/udea.iee.v40n2e11

**Published:** 2022-09-20

**Authors:** Laura Maria Soja Santos, Ana Cristina Wesner, Simone Algeri, Silvana Maria Zarth

**Affiliations:** 1 Nurse Email: lauramssantos31@gmail.com. Federal University of Rio Grande do Sul, Rio Grande do Sul (Brazil) Universidade Federal do Rio Grande do Sul Federal University of Rio Grande do Sul Rio Grande do Sul Brazil lauramssantos31@gmail.com; 2 Nurse, Ph.D. Professor. Email cristinawesner@ufcspa.edu.br: Federal University of Health Sciences of Porto Alegre, Rio Grande do Sul (Brazil) Universidade Federal de Ciências da Saúde de Porto Alegre Federal University of Health Sciences of Porto Alegre Rio Grande do Sul Brazil cristinawesner@ufcspa.edu.br; 3 Nurse, Ph.D. Professor. Email: salgeri@hcpa.edu.br Federal University of Rio Grande do Sul, Rio Grande do Sul (Brazil) Universidade Federal do Rio Grande do Sul Federal University of Rio Grande do Sul Rio Grande do Sul Brazil salgeri@hcpa.edu.br; 4 Nurse, Ph.D. Professor. Email: silvana.zarth@ufrgs.br Federal University of Rio Grande do Sul, Rio Grande do Sul (Brazil) Universidade Federal do Rio Grande do Sul Federal University of Rio Grande do Sul Rio Grande do Sul Brazil silvana.zarth@ufrgs.br

**Keywords:** alert, behavior, knowledge, guilt, education, risk factors, mental health, adolescent health, suicide, sadness., alertas, conducta, conocimiento, culpa, educación, factores de riesgo, salud mental, salud del adolescente, suicidio, tristeza, alerta, comportamento, conhecimento, culpa, educação, fatores de risco, saúde mental, saúde do adolescente, suicídio, tristeza

## Abstract

**Objective.:**

To identify the knowledge of teachers about suicidal behavior in adolescents.

**Methods.:**

Qualitative exploratory-descriptive study conducted in a state school in the municipality of Porto Alegre, RS, Brazil. Twelve teachers participated in the study. Data were collected through semi-structured interviews analyzed using Bardin’s Content Analysis.

**Results.:**

Three categories were built: "Warning signs of suicide", related to the signs identified by the professionals; "Risk factors for suicide", which indicate the reasons that may lead adolescents to present this type of behavior; and "Difficulties in dealing with the behaviors", referring to the behaviors adopted by adolescents and the difficulties of teachers before the theme.

**Conclusion.:**

It was possible to identify that teachers recognize some signs of suicidal behavior, as well as some risk factors. Nonetheless, it is necessary to qualify them to approach the subject, since they feel insecure to act in more critical moments, thus generating mainly feelings of sadness, guilt and powerlessness.

## Resumen

**Objetivo.:**

Identificar el conocimiento de los profesores sobre el comportamiento suicida en adolescentes.

**Métodos.:**

Estudio exploratorio-descriptivo y cualitativo realizado en una escuela pública del municipio de Porto Alegre, RS, Brasil. Doce profesores participaron en el estudio. Los datos se recogieron mediante entrevistas semiestructuradas, que fueron analizadas utilizando el Análisis de Contenido de Bardin.

**Resultados.:**

Se construyeron tres categorías: "Señales de alerta de suicidio", relacionada con las señales identificadas por los profesionales; "Factores de riesgo de suicidio", que indican las razones que pueden llevar a los adolescentes a presentar este tipo de comportamiento; y "Dificultades para lidiar con los comportamientos", relativa a las conductas adoptadas por los adolescentes y a las dificultades de los profesionales frente al tema.

**Conclusión.:**

Se pudo identificar que los profesores reconocen algunas señales de comportamiento suicida, así como algunos factores de riesgo. Sin embargo, es necesario cualificarlas para el abordaje del problema, pues se sienten inseguros para actuar en los momentos más críticos, generando principalmente sentimientos de tristeza, culpa e impotencia.

## Resumo

**Objetivo.:**

Identificar o conhecimento de professores sobre comportamento suicida em adolescentes**.**

**Métodos.:**

Estudo exploratório-descritivo, qualitativo, realizado em uma escola estadual no município de Porto Alegre, RS, Brasil. Participaram no estudo 12 professores. Os dados foram coletados mediante entrevistas semiestruturadas analisadas por meio da Análise de Conteúdo de Bardin.

**Resultados.:**

Foram construídas três categorias: "Sinais de alerta para o suicídio", relacionada com os sinais identificados pelos profissionais; "Fatores de risco para o suicídio", que apontam os motivos que podem levar os adolescentes a apresentarem este tipo de comportamento; e, "Dificuldades em lidar com os comportamentos" referentes às condutas adotadas pelos adolescentes e as dificuldades dos professores frente ao tema.

**Conclusão.:**

Foi possível identificar que os professores reconhecem alguns sinais de comportamento suicida assim como alguns fatores de risco. Porém, é preciso qualificá-los para a abordagem ao assunto, visto que se sentem inseguros para agir em momentos mais críticos, gerando principalmente sentimentos de tristeza, culpa e impotência.

## Introduction

Globally, one in seven young people between 10 and 19 years old has some type of mental disorder, representing 13% of the global burden of disease in this age group. Diseases such as depression and anxiety, as well as behavioral disorders (attention deficit hyperactivity disorder and conduct disorder), are the main causes of comorbidities among adolescents. In turn, suicide is the fourth leading cause of death among young people aged 15 to 19 years.^1^Suicidal behavior comprises suicide ideation, suicide attempt and suicide itself.^2^The increased incidence of suicidal behaviors in adolescence can be explained due to the process experienced by these individuals. Conflicts, changes, physical and sociocultural transformations, which amplify levels of anxiety and depression, are considered risk factors for this type of behavior.^3^ Teachers, because they spend a considerable amount of time with their adolescent students, can be sources of information about signs that indicate their mental health. When qualified, they can help in the identification of risk signs of suicidal behavior occurring at school, being part of the support network in crisis situations.^4^

Given this, the school plays an important role in promoting and protecting the health of students, having a great impact on all aspects of their lives. In this environment, patterns of behavior and relationships that can endanger the health of young people are reproduced.^2^Therefore, the school environment can be a privileged place for early identification of problematic situations and implementation of preventive and protective measures. The nurse who participate in education actions facing intersectoral programs of the public health and education networks must participate in interdisciplinary activities, contributing to the development of skills through training, with the professionals who are closest to the adolescents. In view of this, the objective of the study was to identify the knowledge of teachers about suicidal behavior in adolescents.

## Methods

This is a qualitative exploratory-descriptive study, carried out in a state school in the city of Porto Alegre, RS, Brazil, which serves about 150 children and adolescents between 6 and 17 years old, from the 1^st^ to the 9^th^ grade of elementary school. The choice of the place for the study was by convenience. Twelve teachers who worked in the morning and afternoon shifts were interviewed. The inclusion criteria were: being an active elementary school teacher and being available to participate in the interview. Teachers on Health or Special Leave at the time of data collection were excluded. The collection occurred in the period from July to August 2019, through semi-structured interviews, with open questions, respecting the following themes: perceptions of suicidal behaviors, warning signs of suicide, motivations for suicidal behaviors in adolescents and possible actions in the face of these behaviors and, finally, difficulties before this theme. The interest in the research topic was due to the verification of cases of suicide attempts among adolescents.

The participants were approached face to face and informed that the research would be conducted by an undergraduate nursing student and that the results of this research would be presented in the academic and scientific environment. They were aware of the interview script. As proposed by Gaskell,^5^ the steps for conducting individual interviews were followed. The interviews were carried out by an undergraduate nursing student under the supervision of the person in charge of the research, a PhD in nursing. The researcher developed skills and competencies to conduct interviews during her academic training.

The individual interview was held in a reserved space, with date and time previously arranged, at the workplace of the teacher, during the opposite shift, so as not to interfere with his or her work activities and with a maximum duration of one hour. Only the interviewer and the participant stayed at the interview site, ensuring the accuracy and anonymity of the contained information, omitting the names of the participants and replacing them with numbers. There was no refusal among the educators invited to participate in the research. Similarly, there were no dropouts during the study. Audio recordings were used for data collection, which will be kept for five years under the responsibility of the researchers, and after this period they will be destroyed.There was no need to repeat any interview. Notes were made after reading the interview transcripts thoroughly. The transcripts were not returned to the participants. For data analysis, the Content Analysis method proposed by Bardin was used. It consists of three steps: pre-analysis, exploration of the material and treatment of results, inference, and interpretation.^6^ Data saturation was discussed among the research team members. The researchers transcribed the interviews in Word documents and then coded the data in an Excel spreadsheet, providing a description of the categories that emerged after coding.The categories presented in the research were derived from the obtained data, with quotes from the participants being presented to illustrate the findings according to them. There was consistency between the presented data and the main findings, being clearly presented in the findings, and with discussion of minor themes throughout the research. Due to the onset of the COVID-19 pandemic, the aforementioned institution closed, and then it was not possible to conduct a presentation of the data found in the research to the participants.

The research was approved by the Research Committee of the School of Nursing (COMPESQ/EENF) of the Federal University of Rio Grande do Sul (project number 36425) and by the Research Ethics Committee of the Federal University of Rio Grande do Sul (opinion number 3.416.739), in June 2019. There was also prior authorization from the educational institution in which this research was conducted, with the project being presented and personal objectives and reasons for doing the research clarified. The study participants signed the Free and Informed Consent Form in two copies, one for the interviewee and the other for the researcher.

## Results

The analysis of the interviews allowed the grouping of data into three categories: (1) warning signs, (2) risk factors and (3) actions and difficulties.

### Warning signs

When asked, based on their professional experience, about *"what are the warning signs of suicide?"*, isolation is pointed out as one of the main changes observed in the behavior of adolescents at school, and most of the research participants affirm that they are aware of this change. Likewise, changes in activity or mood levels are reported: *I think that not only isolation, but also too much euphoria. This is also a worrisome thing (E4); She had a very unstable behavior, sometimes she was very depressed and sometimes she was very happy (E8).*

Self-injury is also mentioned as a warning sign, along with a change in the way theadolescent dresses: *Then I asked her: did you do that? She told me: teacher, I feel such a great pain inside me, that I'd prefer to feel physical pain than this pain that I carry inside my chest (E5)*; *[...] she came to talk to me a lot, I noticed that, even in the heat, she was wearing long sleeves and always saying that one day she would end her life. [...] (E11)*. The decrease in school performance is mentioned as a fact that draws the attention of the teacher, believing that something is not right with the student: *He starts getting low grades [...] (E12)*. 

Finally, another important sign reported is suicidal ideation. Adolescents sometimes express suicidal intentions at school, in moments when they are questioned or simply verbalize the desire to commit the act: *I've already had cases of students who came to me and verbalized: "I don't want to live anymore" (I12), They say: "I'm going to kill myself," and we think it's a child and it won't happen ...(E11).*

### Risk factors

When asked about possible risk factors for the development of suicidal behaviors, some interviewees bring up statements that relate them to the issue of body acceptance and low self-esteem: *I think that acceptance, that standard thing, I'm too fat, I can't have my group of friends because no one will accept me or I'm too thin (E1); There's another boy who was fat, he's still fat, but he got breasts, so he really wants to have that fat removed. Then, the question of acceptance arises (E6);* The bullying that can occur in the school environment appears in the reports as a reason for concern for professionals*; There are students who are so terrified of this type of aggression from their peers (bullying) that they end up thinking that they are not part of society because they are not equal to the standards they have set in their heads. (E5).*

The use of the internet is mentioned as an important element that requires attention, because there is too easy access to social and digital media, which can generate negative influences on the adolescent: *Not to mention the internet. And since they are vulnerable, they are ashamed of everything, "what do people think of me?", exposure is one thing ... (E8); People, our own students, see a lot of things on social networks. Remember there was a program on social networks that said "do it", or "if you don't do it, kill, you have to do it and kill". Blue whale (E7);*Some teachers point out the occurrence of psychiatric disorders, especially depression, in the development of suicidal behaviors*. I understand that this suicidal behavior starts with a depression [...] (E1); Because of a depression, I attempted suicide all the time (E12).*

The lack of family structure, often associated with the occurrence of domestic violence, alcohol and drug use, as well as being in a situation of social vulnerability, has already alerted the professionals who live with these adolescents: *First of all it is the family, the family disorganization can be a risk factor (E8); They have these social issues we can say so that are quite aggravating, the lack of resources, family, the environment where they are, their social vulnerability situation (E12);One factor that can lead to suicide is domestic violence (E12); A student told me the other day that his mother drinks, his father drinks, his grandfather drinks and he drank too. He told me that his stepfather allows him to drink beer. I told him that it is wrong. Then you understand children; usually they are innocent and are portraying what they see inside the home. It is complicated (I1).*

### Actions and difficulties

For teachers, when there is a suspicion of suicidal behavior, the most common approach is to seek guidance from the Administration or Educational Guidance Service (SOE, as per its Portuguese acronym) of the school, according to the statements below: *Well, first place I would seek help at the school, at the SOE, with the Administration, seek support in what I have within the school [...] We are a team and we have to help each other. Accordingly, with them, we have to look for what we can do, look for our rights (E2); I would talk to the manager, with the SOE [...] it's hard (E7).*

Some reports talk about the importance of communicating with the family: *Look, as I told you; it is important to call the parents urgently, report the fact (E5); After (passing the case to the administration) we call the family member to be able to talk (E9).* According to the statements, it is necessary to follow-up the outcome of the case, because if family negligence is identified, other directions can be taken: *And then there is that, pass it on to the Administration, to the Guardianship Council; these are the means we have (E10); If the Guardianship Council can't solve it, then we go to the Public Prosecutor's Office. That’s the way (E11).*

In turn, the reported lack of preparation leads to difficulties in dealing with the situation, thus generating feelings of guilt, for not having been able to identify something before; and of sadness, for believing that they cannot help enough: *We do much more than we should, not because we don't want to, but we are not prepared. I was very afraid, even when I wrote a letter to a student who was hospitalized for self-injury. I was afraid of what I could or couldn't say, we don't have guidance, we don't have preparation on the subject (E8).*

The speeches show the internal conflict on the part of teachers when faced with a student presenting suicidal behavior. Conducts adopted with insecurity lead them to worry that they are doing something wrong or that may worsen the situation of an adolescent. In the attempt to help, they also end up suffering:*Gosh! Something happens in class, tomorrow the guy doesn't come. Two days later you find out that he tried to commit suicide. Therefore, you feel guilty because a word you could have said! (E1);When I got his essay I burst into tears, I started to cry, cry, cry. Then the manager got worried, "this kid is going to kill himself, we have to do something. I was very shaken psychologically (E9); I did what I thought was right at the time, but today I think I was a little negligent, I think I should have called before, should have sent for the father before, because I tried to talk to her*(manager)*, you know, not to expose this to the father because we do not know the depth of the thing (E8).*

The overload of work and the concern of the teacher with literacy were also cited as difficulties related to the perception of signs consistent with suicidal behavior. Together with the lack of information to adapt them to the subject, these factors can lead teachers to not notice situations that are happening with the student in the classroom. The narrated experiences are below: *But they signal, we,due to the rush of day to day, don't stay so attentive. (E11) With children, sometimes we are worried about teaching literacy, discipline and behavior, and sometimes it goes unnoticed. If it's a larger group, it goes unnoticed; if they are fighting, one at the other's desk, sometimes this can go unnoticed (E3).*

Even with the qualification of the teachers, it is still necessary to improve sensitivity and empathy to act when faced with a critical situation. The statements corroborate these as important strategies in what concerns suicide prevention: *I'm always taking care, I'm attentive to my students, I always try to know more, how they act outside school, how they are treated, I get into these conversations to understand a little better what is going on (E5); I'm doing some dynamics with them.I enter the room to work on issues of emotion, but I feel that there is a lack of theoretical basis to better understand the drawings, the meanings, and here we have openness to do so (E4).* The participants emphasize the importance of being alert to disrespectful behaviors among adolescents and willing to talk openly about respect and individuality*: [...] when the student comes from another school and calls our attention, many are not educated to deal with differences and start joking when they should not.I said "no, everyone has to respect each other, no one is programmed to have a standard, they will seek that throughout life and we, as human beings, have to respect it", this is the message I pass on to them (E5).*

As the following statement shows, the participants understand the need to address the issue in the school environment, so that young people can feel safe to share their feelings. *I always try to talk to them in class, I'm very honest with them, I tell them that I've had depression, serious problems, that I still take medicines, that they have to be honest about it, for them to talk to their parents, talk to us, to an older friend, always something for them to seek help, pay attention to their friends (E8).*

## Discussion

This study sought to identify what teachers know about suicidal behavior in adolescents and how they face this situation at school. Isolation, along with mood swings, were indicated as the main changes observed by teachers. The literature describes isolation and mood swings as warning signs of suicidal behavior, along with the practice of self-injury.^2^,^7^Corroborating with these findings, Gijzen et al, using a large community sample of adolescents aged 11 to 16, found that loneliness was a central factor for depression networks and also the factor that contributed most to suicidal ideation.^8^ Regarding mood swings, one study demonstrated that transient impulsive choice abnormalities are found in a subset of those who attempt suicide.Both suicidal ideation and behavior were associated with impulsivity of choice and intense psychological pain.^9^Regarding emotional dysregulation, a study conducted with adolescent girls with borderline personality disorder identified that participants who had greater difficulty in regulating their emotional experiences were at greater risk for making a suicide attempt.^10^

Self-injuries in adolescents were also identified by teachers in the school environment. Data from the Brazilian Ministry of Health point out that, nationally, the age group between 10 and 19 years old appears in second place in the occurrences of self-injury, and this practice is considered a predictive factor for future suicide attempts.^11^,^12^ According to this result, studies show that the main risk factors for self-injury include bullying, concomitant mental illness and a history of childhood abuse and neglect.^12^,^13^ Decreased school performance also appears as a major change in attitudes among adolescents.^2^,^14^This event may be associated with depression, characterized by a series of signs and symptoms that go beyond a decline in performance at school, such as irritability/instability, difficulty concentrating, feelings of hopelessness and/or guilt, sleep alterations, suicidal ideation and suicide attempts. Suicidal ideation is directly related to most symptoms of depression in adolescents.^8^,^15^ Results from one study show that anxiety and suicide are strongly linked, since anxiety amplifies the stress response, thus increasing suicidal tendencies.^16^

Regarding the risk factors cited by the participants, they bring issues related to body acceptance and low self-esteem. Adolescents dissatisfied with their image are, in fact, more susceptible to suicidal ideation, as shown in the literature.^2^,^3^ Bullying also appears as a risk factor, since suicidal behavior in the studied age group is several times related to these acts of violence.^2^When talking about bullying, it is important to think not only about the victims, but also about the perpetrators, because both suffer emotional and social consequences. Nonetheless, the anguish generated in this process is responsible for the development of depressive symptoms, especially in the victims.^17^

According to UNICEF data, cyberbullying also affects young people. In Brazil, 37% of them say they have already been victims of the practice, and 36% report that they have stopped going to school after online violence. Because of this, social and digital media can also influence the development of suicidal behaviors.^2^,^18^The results of one study showed that bullying, cyberbullying and peer problems are related, thus indicating a direct and positive relationship with suicidal behavior.^19^As far as family relationships are concerned, they can assume the role of risk or protective factors. Difficulties in relationships, communication, lack of affection and support, fights, physical and verbal violence are present in dysfunctional family relationships. Being exposed to an environment in which young people experience displacement and a sense of not belonging contributes to the occurrence of suicidal behavior in adolescents.^14^,^20^

On the other hand, there are studies that show that satisfaction in interpersonal relationships, especially with the family, is a very important protective factor for suicide attempts, since the offered family support has the potential to decrease the psychological impact of difficult situations faced by the individual.^20^A review study identified that the most common protective factors for both suicide and bullying were being female, having good mental health, belonging to a two-parent family, safe school environment, good family relationships and having an involved teacher.^21^The use of alcohol and drugs also presents itself as a risk factor, as shown by Neto and Pelizzari, who, after researching the medical records of young people followed-up in a Psychosocial Care Center for Alcohol and Other Drugs (CAPS AD, as per itis Portuguese acronym), with ages between 12 and 18 years, found that 97% of the records analyzed with complaints of suicide attempts were related to alcoholic ingestion. The use of drugs such as marijuana and cocaine, practice of self-injury and being female are also cited as risk factors by the authors.^22^In this sense, evidence identifies that social vulnerability in adolescents is related to negative aspects, especially those associated with involvement with drugs, loss of guaranteed rights and opportunities in the areas of education, health and social protection, with situations of violence, whether domestic or communal, and child labor.^19^

Despite still being treated as taboo, issues related to psychological suffering and mental disorders, especially in the adolescent phase, have been taking place in the school environment and require rapid and early interventions, in order to prevent these young people from developing risk behaviors that culminate in suicide. For example, a multicenter European study, which conducted an intervention entitled Young Mental Health Aware Program, which aimed to prevent suicide at school for students at risk.The program was effective in reducing the number of suicide attempts and severe suicidal ideation in school adolescents in the long term (12 months).^23^ Thus, considering the role of the school in the lives of children and adolescents, it is evident that this is a privileged environment for the promotion of mental health and suicide prevention. The insertion of suicide prevention strategies in schools is necessary.

During the study, it is evident that educators lack contact with the theme during their training and while working in schools, a fact that can be observed through the reports about the difficulty in acting when faced with a case of suicidal behavior. Concomitantly with this research, another similar study shows that teachers are unaware of the warning signs of suicidal behavior among students. They also report that they do not know how to support students in case of an attempt or completed suicide of another student. Reinforcing the data found in the current research, it was also pointed out that the school curriculum is perceived as lacking information about suicide and suicidal behavior.^24^

The action taken by the teacher shows the concern of this professional for the theme and, in particular, the concern for his or her student as a human being who, sometimes, needs to feel safe to be heard, truly heard. Considered a public health problem,^1^ suicide requires that nurses inserted in the context of health education and through the School Health Program^25^promote the strengthening of bonds between family, students and teachers, thus creating spaces for discussion to systematize, reflect and organize intersectoral work, in order to overcome the difficulties related to the subject under discussion.The lack of understanding about the subject generates emotional discomfort for all involved, and it is up to the health professional to welcome this demand, whether by the adolescents or teachers, and assist in the prevention and promotion of health.

The limitations of the study were due to the number of participants and the fact that the research was carried out in a single center, which does not allow the results to be generalized. There is a need to expand the studies on the work of nurses in school contexts, in order to be able to assist professionals in the field of education in the early identification of potential cases of suicide. However, this study can serve as a basis for new studies, and the results can be used to improve health care in school, prevent suicide in adolescents, and/or support policies aimed at achieving these goals.

## Conclusion

It was possible to identify that teachers recognize some signs of suicidal behavior, as well as some risk factors. Nonetheless, it is necessary to qualify them to approach the subject, since they feel insecure to act in more critical moments, thus generating mainly feelings of sadness, guilt and powerlessness.
